# Autoimmunity associates with severity of illness in elderly patients with drug-induced liver injury

**DOI:** 10.3389/fphar.2023.1071709

**Published:** 2023-02-16

**Authors:** Yu-Ting Xiong, Jian-Fei Wang, Xiao-Xia Niu, Yi-Ming Fu, Ke-Xin Wang, Chun-Yan Wang, Qian-Qian Li, Jian-Jun Wang, Jun Zhao, Dong Ji

**Affiliations:** ^1^ Department of Hepatology, Fifth Medical Center of Chinese PLA General Hospital, Beijing, China; ^2^ 307 Clinical Medical College of PLA, Anhui Medical University, Beijing, China; ^3^ Emergency Department, Seventh Medical Center of Chinese PLA General Hospital, Beijing, China; ^4^ Chinese PLA Medical School, Beijing, China

**Keywords:** drug-induced liver injury (DILI), elderly, hepatic fibrosis, autoimmunity, liver biopsy

## Abstract

**Background:** Drug-induced liver injury (DILI) is a potentially serious adverse drug reaction. Due to the lack of definite etiology, specific clinical manifestations, and diagnostic methods, its prediction and diagnosis are challenging. Elderly individuals are deemed to be at high risk for DILI due to abnormal pharmacokinetics, aging tissue repair function, comorbidities, and taking multiple drugs. This study aimed to identify the clinical characteristics and explore the risk factors associated with the severity of illness in elderly patients with DILI.

**Methods:** In the present study, the clinical characteristics at the time of liver biopsy of consecutive patients with biopsy-proven DILI who presented at our hospital from June 2005 to September 2022 were evaluated. Hepatic inflammation and fibrosis were assessed according to the Scheuer scoring system. The presence of autoimmunity was considered if IgG level >1.1 × ULN (1826 mg/dL), or high titer (>1:80) of ANA, or SMA.

**Results:** In total, 441 patients were enrolled, and the median age was 63.3 years (IQR, 61.0–66.0); 122 (27.7%), 195 (44.2%), or 124 (28.1%) were classified as having minor, moderate, or severe hepatic inflammation, respectively; and 188 (42.6%), 210 (47.6%) or 43 (9.8%) patients presented minor, significant fibrosis or cirrhosis, respectively. Female sex (73.5%) and the cholestatic pattern (47.6%) were dominant in elderly DILI patients. Autoimmunity existed in 201 patients (45.6%). Comorbidities were not directly associated with the severity of DILI. PLT (OR: 0.994, 95% CI: 0.991–0.997; *p* < 0.001), AST (OR: 1.001, 95% CI: 1.000–1.003, *p* = 0.012), TBIL (OR: 1.006, 95% CI: 1.003–1.010, *p* < 0.001), and autoimmunity (OR: 1.831, 95% CI: 1.258–2.672, *p* = 0.002) were associated with the degree of hepatic inflammation. Meanwhile, PLT (OR: 0.990, 95% CI: 0.986–0.993, *p* < 0.001), TBIL (OR: 1.004, 95% CI: 1.000–1.007, *p* = 0.028), age (OR: 1.123, 95% CI: 1.067–1.183, *p* < 0.001), and autoimmunity (OR: 1.760, 95% CI: 1.191–2.608, *p* = 0.005) were associated with the stage of hepatic fibrosis.

**Conclusion:** This study revealed that the presence of autoimmunity represents a more serious illness state of DILI, deserving more intensive monitoring and progressive treatment.

## Introduction

In the case of a reasonable rule out of other causes, drug-induced liver injury (DILI), which remains one of the most challenging diseases faced by hepatologists, is a severe adverse drug reaction caused by hepatotoxic exogenous agents and their metabolites, such as prescriptions, over-the-counter drugs, herbs, and dietary supplements (Teschke et al., 2013; Kleiner et al., 2014; Navarro et al., 2014; EASL-ALEH Clinical Practice Guidelines;Asociacion Latinoamericana para el Estudio del Higado, 2015; Foureau et al., 2015; Bonkovsky et al., 2017; Kullak-Ublick et al., 2017; Andrade et al., 2019b). With the enhancement of people’s health consciousness, herbal and dietary supplements (HDS) or traditional Chinese medicines (TCM) account for an increasing proportion of DILI events worldwide (Wai et al., 2007; Navarro et al., 2014; García-Cortés et al., 2016; Ji et al., 2018). This is especially applicable to body-building and fat-reducing supplements (Grewal and Ahmad, 2019; Santos et al., 2021), exposing people to more uncertain hepatotoxic drugs, which calls for clinicians to have a greater understanding of DILI.

Elderly individuals are at high risk for DILI (Danan and Benichou, 1993; Lucena et al., 2009; Andrade et al., 2019a). First, the distribution and release of drugs are affected by increased body fat in the aged (Lucena et al., 2009). Second, the decrease in liver and kidney function affects the metabolism and excretion of drugs, leading to abnormal pharmacokinetics (Tostmann et al., 2008; Klotz, 2009; Andrade et al., 2019a). At the same time, their tissue-repair capacity is reduced. In addition, the aged have many comorbidities and may require multiple medications, so DILI is complicated by drug-drug interactions and drug-host interactions (Chen et al., 2015; Andrade et al., 2019a). Marcum et al. (2012) reported that people older than 70 take an average of three to seven medications per day. A meta-analysis demonstrated that the prevalence of polypharmacy (using more than five drugs) and potentially inappropriate medications (PIM) in elderly Chinese patients was 48% and 39%, respectively (Tian et al., 2022). Similarly, Koçak et al. (2022) mentioned that the prevalence of PIM among nursing home residents aged ≥60 years was 47.6%.

DILI with features of autoimmunity represents an important category of hepatotoxicity due to medication exposure, which is a syndrome typically characterized by liver injury accompanied by hypergammaglobulinemia, circulating anti-nuclear antibody (ANA) and/or smooth muscle antibody (SMA) (deLemos et al., 2014). Circulating antibodies targeting intracellular components are also indicative of cellular damage. Furthermore, elderly individuals commonly present a low titer (<1:80) of serum autoantibodies (approximately 25%), making distinguishing between DILI and AIH more complicated (deLemos et al., 2014).

The exclusive diagnosis combined with causality assessment is an essential and arduous task to improve DILI diagnosis (Liu et al., 2021). There is considerable variability in the time to onset, severity, clinical manifestations, laboratory features, findings on liver biopsy, course, and outcome. Moreover, relatively few studies have involved elderly DILI patients with features of autoimmunity, making the database for this group somewhat limited. Thus, in the present study, we aimed to identify the clinical characteristics and explore the risk factors associated with the severity of illness in this special patient population to avoid progressing to poorer clinical outcomes.

## Patients and methods

### Study design and patients

The present study was a retrospective hospitalization-based cross-sectional study, which was approved by the Ethics Committees of Fifth Medical Center of Chinese PLA General Hospital (No. 2019024D). Written informed consent for liver biopsy was obtained from all patients, whereas patient consent for data collection was waived due to the study design.

The inclusion criteria included the following: 1) admitted from June 2005 to September 2022; 2) age ≥60 years old; 3) met the DILI definition (see definition section); 4) Roussel Uclaf Causality Assessment Method (RUCAM) score >6 points; and 5) the diagnosis of DILI was confirmed by liver biopsy. The exclusion criteria were as follows: 1) those with any other definite etiologies of liver disease (e.g., primary biliary cholangitis, primary sclerosing cholangitis, viral hepatitis, alcoholic or non-alcoholic liver disease, Gilbert syndrome, etc.) according to their relevant guidelines (Lindor et al., 2015; EASL–EASD–EASO Clinical Practice, European Association for the Study of Diabetes EASD, European Association for the Study of Obesity EASO, 2016; Hirschfield et al., 2017; Singal et al., 2018; Wagner et al., 2018; Te and Doucette, 2019); 2) those with severe systemic diseases affecting the liver (heart attack, stroke, kidney, or HIV infection); and 3) those with incomplete important data.

### Procedures

The clinical data of the enrolled patients at the time of liver biopsy were retrieved through electronic medical records, such as age, sex, body mass index (BMI), implicated drugs, platelet (PLT), alanine aminotransferase (ALT), aspartate aminotransferase (AST), total bilirubin (TBIL), alkaline phosphatase (ALP), immunoglobulin G (IgG), anti-nuclear antibody (ANA), smooth muscle antibody (SMA), and anti-mitochondrial antibody (AMA).

### Definition

DILI was defined as an adverse hepatic reaction that is unexpected based on the pharmacological action of the drug administered, including one of the following thresholds: 1) ≥5 × ULN elevation in ALT, 2) ≥2 × ULN elevation in ALP (particularly with accompanying elevations in concentrations of GGT in the absence of known bone pathology driving the rise in ALP level), or 3) ≥3 × ULN elevation in ALT and simultaneous elevation of TBIL concentration exceeding 2 × ULN, according to EASL DILI guideline 2019. Three patterns of DILI were categorized by using the R-value ([ALT/upper limit of the normal range (ULN)]/[ALP/ULN]): hepatocellular when ≥5, cholestatic when ≤2 and mixed when 2–5 (Andrade et al., 2019a).

The presence of autoimmunity was considered if IgG level >1.1 × ULN (1826 mg/dL), or high titer (>1:80) of ANA, or SMA (de Boer et al., 2017). The enrolled patients were divided into three groups by age: Group A, ≤65 years (*n* = 318 [72.1%]); Group B, 65–70 years (*n* = 92 [20.9%]); and Group C, >70 years (*n* = 31 [7.0%]).

### Histological evaluations

Ultrasound-guided liver biopsy was performed on all patients, and hepatic inflammatory grades and fibrosis stages were evaluated according to the Scheuer scoring system (Scheuer, 1991). To ensure sufficient specimens with at least 12 portal vessels for histological evaluation, a minimum length of 15.0 mm was required for each liver specimen. Subsequently, two liver histopathologists independently evaluated tissue specimens, and when inconsistencies arose, both pathologists re-reviewed the specimens together. The grade of hepatic inflammation was defined as mild (G0-1), moderate (G2), and severe (G3-4). The stage of hepatic fibrosis was defined as mild (S0-1), significant (S2-3), and cirrhosis (S4).

### Statistical analysis

Continuous variables were expressed as medians and interquartile ranges (IQR) and compared by using the Kruskal-Wallis test. Categorical variables were presented as numbers (percentage) and compared by Chi-square or Fisher’s exact test. The trend was analyzed by the Cochran-Armitage trend test for the 2 × 3 tables (sex and autoimmunity) and the Jonckheere-Terpstra trend test for the 3 × 3 tables (age, liver damage pattern, medication). Multivariate ordinal polytomous logistic regression was performed to identify the independent risk factors associated with the severity of illness regarding hepatic inflammation or fibrosis. The odds ratio (OR) and 95% confidence interval (CI) were estimated simultaneously. The forest plot was established by using the ggplot2 package of R. A two-tailed *p-value* of <0.05 was considered statistically significant. All statistical analyses were performed using R software, version 4.2.1 (http://www.r-project.org/).

## Results

### Clinical characteristics

**FIGURE 1:**
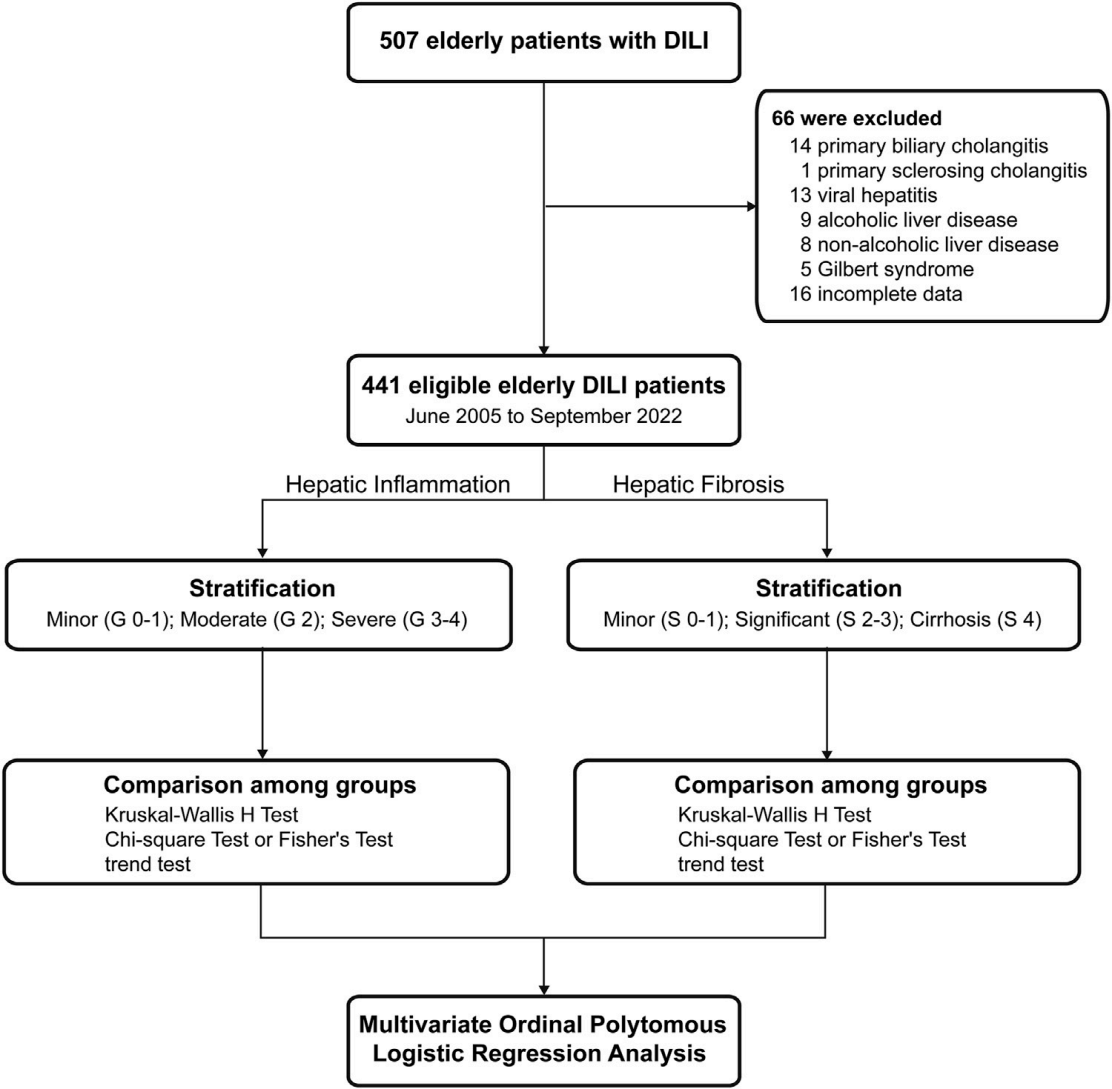
Flow chart of study population. DILI, drug-induced liver injury.

A total of 507 elderly patients with DILI who underwent liver biopsy were screened. Of these patients, 66 patients were excluded. Finally, 441 patients were enrolled in this study and were categorized into three groups according to hepatic inflammation grade or fibrosis stage (Figure 1).

The overall median age was 63.3 years (IQR, 61.0–66.0), and 324 patients (73.5%) were female. Regarding histological characteristics, 122 (27.7%), 195 (44.2%), or 124 (28.1%) patients were classified as having minor, moderate, or severe hepatic inflammation, respectively; 188 (42.6%), 210 (47.6%), or 43 (9.8%) patients presented minor, significant fibrosis, or cirrhosis, respectively. The clinical pattern of liver damage was given priority to cholestatic damage (47.6%), followed by hepatocellular damage (29.3%), and mixed damage (23.1%). The most common comorbidities were hypertension (28.1%), hyperlipidemia (27.7%), diabetes mellitus (11.8%), and coronary heart disease (4.8%). According to the results of autoimmune antibody detection, there were 143 patients (32.4%) with positive ANA, 30 patients (6.8%) with positive SMA, and 74 (16.8%) with ≥1.1 × ULN of IgG. Overall, 201 patients (45.6%) presented the feature of autoimmunity (Table 1).

**TABLE 1:** Clinical characteristics stratified by hepatic inflammation.

	Overall (*N* = 441)	Minor (*N* = 122)	Moderate (*N* = 195)	Severe (N = 124)	*p*-value
Age (years)	63.3 (61.0–66.0)	62.0 (61.0–65.0)	64.0 (61.1–66.0)	63.3 (61.0–66.0)	0.191
Age groups					0.916
≤65 years	318 (72.1)	92 (75.4)	138 (70.8)	88 (71.0)	
65–70 years	92 (20.9)	22 (18.0)	43 (22.1)	27 (21.8)	
>70 years	31 (7.0)	8 (6.6)	14 (7.2)	9 (7.3)	
Female sex	324 (73.5)	85 (69.7)	140 (71.8)	99 (79.8)	0.152
BMI (kg/m^2^)	23.8 (22.2–25.6)	23.5 (22.4–25.0)	24.0 (22.5–25.6)	23.8 (21.8–26.1)	0.276
Hypertension	124 (28.1)	34 (27.9)	58 (29.7)	32 (25.8)	0.746
Hyperlipidemia	122 (27.7)	39 (32.0)	49 (25.1)	34 (27.4)	0.415
Diabetes mellitus	52 (11.8)	11 (9.0)	27 (13.8)	14 (11.3)	0.422
Coronary heart disease	21 (4.8)	5 (4.1)	8 (4.1)	8 (6.5)	0.581
Class of Implicated drugs					0.790
TCM or HDS	110 (24.9)	27 (22.1)	47 (24.1)	36 (29.0)	
Synthetic drugs	129 (29.3)	37 (30.3)	58 (29.7)	34 (27.4)	
Combined	202 (45.8)	58 (47.5)	90 (46.2)	54 (43.5)	
Pattern of liver damage					<0.001
Hepatocellular	129 (29.3)	17 (13.9)	62 (31.8)	50 (40.3)	
Cholestatic	210 (47.6)	81 (66.4)	88 (45.1)	41 (33.1)	
Mixed	102 (23.1)	24 (19.7)	45 (23.1)	33 (26.6)	
PLT (×10^9^/L)	170.0 (127.0–209.0)	185.0 (136.5–232.5)	170.0 (122.5–206.0)	154.0 (122.8–190.8)	0.009
ALT (U/L)	76.0 (35.0–203.0)	36.0 (22.0–96.2)	89.0 (42.5–246.5)	124.5 (53.0–333.5)	<0.001
AST (U/L)	72.0 (38.0–183.0)	32.5 (25.2–55.5)	80.0 (44.5–181.0)	132.5 (80.5–324.0)	<0.001
TBIL (μmol/L)	18.2 (11.0–37.3)	11.5 (8.7–17.3)	18.6 (11.6–35.9)	35.4 (17.1–80.5)	<0.001
ALP (U/L)	130.0 (97.0–179.0)	114.0 (79.2–156.0)	134.0 (101.5–181.5)	138.5 (103.0–180.5)	0.001
IgG ≥1.1 × ULN	74 (16.8)	8 (6.6)	32 (16.4)	34 (27.4)	<0.001
ANA positive	143 (32.4)	33 (27.0)	60 (30.8)	50 (40.3)	0.068
SMA positive	30 (6.8)	4 (3.3)	12 (6.2)	14 (11.3)	0.040
Autoimmunity	201 (45.6)	43 (35.2)	83 (42.6)	75 (60.5)	<0.001

ALT, alanine aminotransferase; AST, aspartate aminotransferase; ALP, alkaline phosphatase; ANA, anti-nuclear antibody; AMA, anti-mitochondrial antibody; BMI, body mass index; HDS, herbal and dietary supplements; IgG, immunoglobulin G; PLT, platelet; SMA, smooth muscle antibody; TBIL, total bilirubin; TCM, traditional Chinese medicine; ULN, upper limits of normal.

**FIGURE 2:**
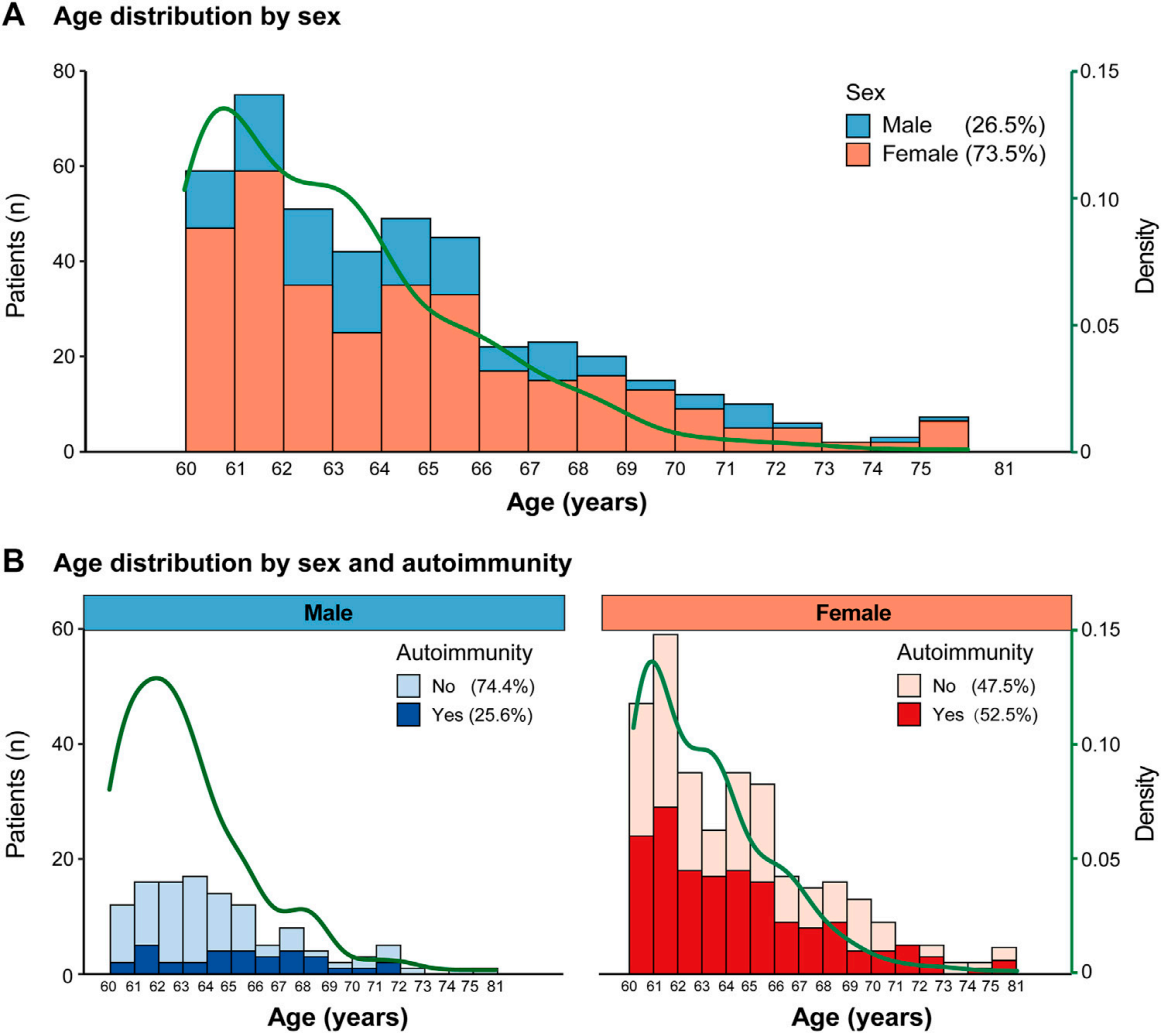
Age distribution **(A)**. Age distribution by sex; **(B)**. Age distribution by sex and autoimmunity.

Furthermore, we investigated the distribution of age stratified by sex. The number of cases was highest among those aged 60–62 years, and the percentage of females was significantly higher than that of males for every 1-year age group (*p* < 0.05, Figure 2A). The overall percentage of autoimmunity in females was 52.5%, which was significantly higher than that in males (25.6%, *p* < 0.05, Figure 2B).

### Implicated drugs

In total, 202 (45.8%) patients had taken a combination of synthetic drugs and TCM or HDS, 129 (29.3%) had taken synthetic drugs, and 110 (24.9%) had taken TCM or HDS. The most commonly implicated drugs were cardiovascular drugs (17.0%), followed by herbal and dietary supplements (HDS) (12.9%), gastrointestinal drugs (6.3%), non-steroidal anti-inflammatory drugs (NSAIDs) (5.9%), etc. Meanwhile, there were 16.3% polypharmacy and 21.5% unspecified drugs (Supplementary Figure S1).

### Distribution of clinical Parameters by histological assessment

**FIGURE 3:**
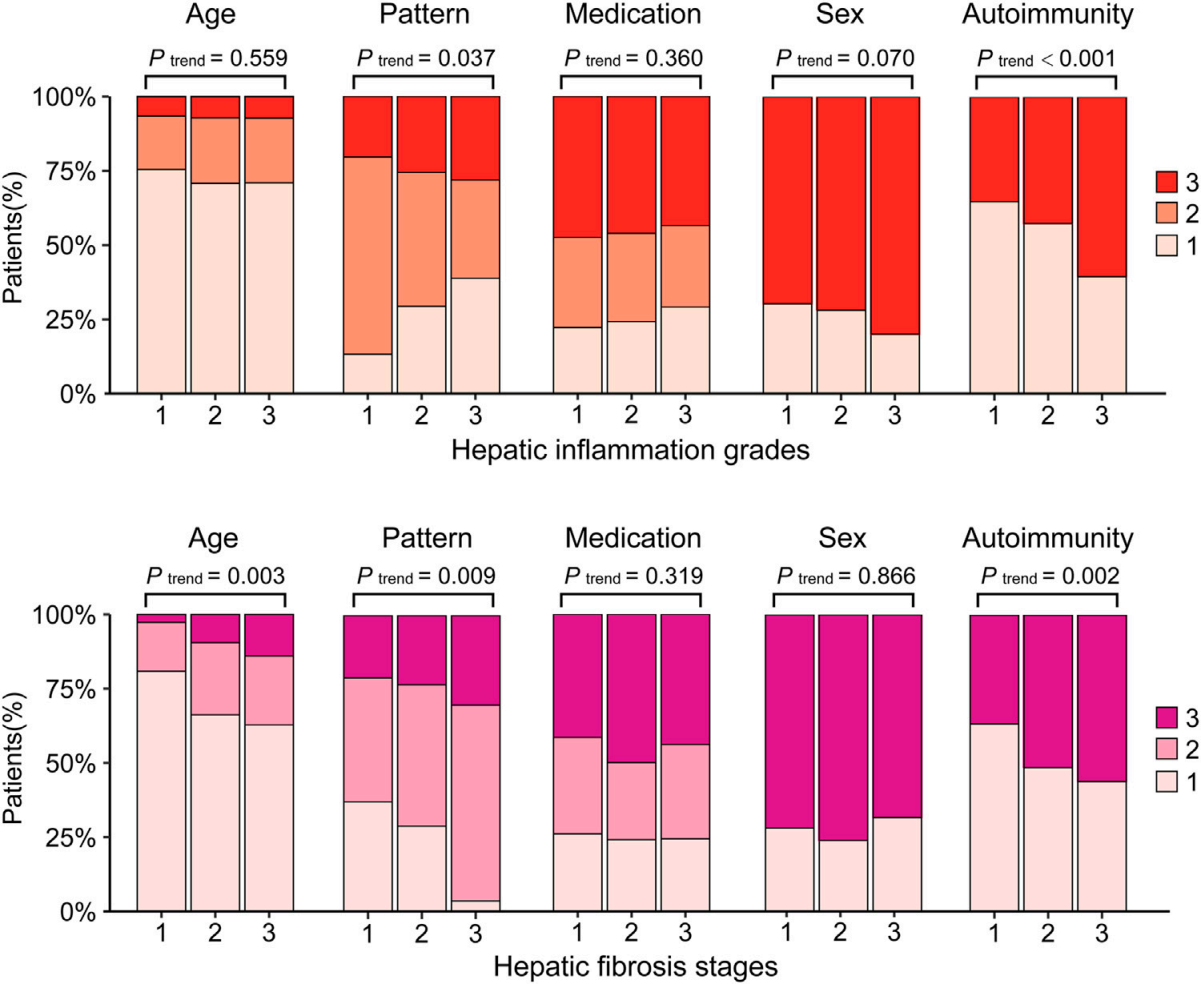
The clinical characteristics by histological evaluation.Hepatic inflammation grade: 1: G0-1; 2: G2; 3: G3-4. Hepatic fibrosis stage: 1: S0-1, 2: S2-3, 3: S4. Age, 1: ≤65 years, 2: 65–70 years, 3: >70 years. Pattern, 1: hepatocellular; 2: Cholestatic; 3: Mixed. Medication, 1: Synthetic drugs; 2: Traditional Chinese Medicine or herbal and dietary supplements; 3: Combined. Sex, 1: Male; 3: Female. Autoimmunity: 1: No; 3: Yes.

All enrolled patients were categorized into three ordinal groups according to histological assessment and compared. The results showed that patterns of liver damage, PLT, ALT, AST, TBIL, and autoimmunity were significantly different in groups with increasing grades of hepatic inflammation (*p* < 0.05, Table 1) or in groups with increasing stages of hepatic fibrosis (*p* < 0.05, Table 2). Furthermore, with the aggravation of hepatic inflammation grade, the proportion of hepatocellular pattern (13.9%, 31.8%, and 40.3%, *p* for trend = 0.037) and presence of autoimmunity (35.2%, 42.6%, and 60.5%, *p* for trend <0.001) increased significantly. Similarly, with the progression of hepatic fibrosis stage, the proportion of older age >70 years (2.7%, 9.5%, and 14.0%, *p* for trend = 0.003), cholestatic pattern (43.6%, 47.6%, and 65.1%, *p* for trend = 0.009), and presence of autoimmunity (36.7%, 51.0%, and 58.1%, *p* for trend = 0.002) significantly increased (Figure 3).

**TABLE 2:** Clinical characteristics stratified by hepatic fibrosis.

	Minor (*N* = 188)	Significant (*N* = 210)	Cirrhosis (*N* = 43)	*p*-value
Age (years)	62.2 (61.0–65.0)	64.0 (61.1–67.0)	63.4 (61.6–67.9)	0.002
Age group				0.002
≤65 years	152 (80.9)	139 (66.2)	27 (62.8)	
65–70 years	31 (16.5)	51 (24.3)	10 (23.3)	
>70 years	5 (2.7)	20 (9.5)	6 (14.0)	
Female sex	135 (71.8)	159 (75.7)	30 (69.8)	0.574
BMI (kg/m^2^)	23.5 (22.0–25.1)	24.0 (22.3–26.0)	24.2 (22.6–25.2)	0.206
Hyperlipidemia	19 (10.1)	25 (11.9)	8 (18.6)	0.296
Hypertension	43 (22.9)	73 (34.8)	8 (18.6)	0.011
Diabetes mellitus	7 (3.7)	12 (5.7)	2 (4.7)	0.648
Coronary heart disease	55 (29.3)	57 (27.1)	10 (23.3)	0.710
Class of Implicated drugs				0.570
TCM or HDS	49 (26.1)	51 (24.3)	10 (23.3)	
Synthetic drugs	61 (32.4)	55 (26.2)	13 (30.2)	
Combined	78 (41.5)	104 (49.5)	20 (46.5)	
Pattern of liver damage				0.001
Hepatocellular	66 (35.1)	62 (29.5)	1 (2.3)	
Cholestatic	82 (43.6)	100 (47.6)	28 (65.1)	
Mixed	40 (21.3)	48 (22.9)	14 (32.6)	
PLT (×10^9^/L)	184.0 (149.5–234.2)	157.5 (118.2–196.8)	117.0 (93.5–162.0)	<0.001
ALT (U/L)	99.5 (34.5–253.8)	76.5 (38.0–209.5)	52.0 (31.0–66.5)	0.006
AST (U/L)	61.0 (31.8–176.2)	91.5 (45.5–224.8)	63.0 (43.5–95.0)	0.005
TBIL (μmol/L)	15.1 (9.8–32.3)	22.0 (12.1–42.9)	15.6 (11.3–24.4)	0.002
ALP (U/L)	123.5 (91.0–178.0)	138.0 (103.0–182.0)	120.0 (93.5–171.5)	0.086
IgG ≥ 1.1 × ULN	16 (10.0)	45 (25.0)	13 (41.9)	<0.001
ANA positive	46 (24.5)	79 (37.6)	18 (41.9)	0.008
SMA positive	9 (4.8)	16 (7.6)	5 (11.6)	0.223
Autoimmunity	69 (36.7)	107 (51.0)	25 (58.1)	0.004

ALT, alanine aminotransferase; AST, aspartate aminotransferase; ALP, alkaline phosphatase; ANA, anti-nuclear antibody; BMI, body mass index; HDS, herbal and dietary supplements; IgG, immunoglobulin G; PLT, platelet; SMA, smooth muscle antibody; TBIL, total bilirubin; TCM, traditional Chinese medicine; ULN, upper limits of normal.

### Multivariate ordinal polytomous logistic regression analysis

After screening by comparison among groups, collinearity analysis, and ordinal univariate analysis, variables with a *p-*value of <0.1 were included in the multivariate ordinal polytomous logistic regression analysis (Table 3). Ultimately, PLT (OR: 0.994, 95% CI: 0.991–0.997; *p* < 0.001), AST (OR: 1.001, 95% CI: 1.000–1.003, *p* = 0.012), TBIL (OR: 1.006, 95% CI: 1.003–1.010, *p* < 0.001), and autoimmunity (OR: 1.831, 95% CI: 1.258–2.672, *p* = 0.002) were associated with the degree of hepatic inflammation. Meanwhile, PLT (OR: 0.990, 95% CI: 0.986–0.993, *p* < 0.001), TBIL (OR: 1.004, 95% CI: 1.000–1.007, *p* = 0.028), age (OR: 1.123, 95% CI: 1.067–1.183, *p* < 0.001), and autoimmunity (OR: 1.760, 95% CI: 1.191–2.608, *p* = 0.005) were associated with the stage of hepatic fibrosis.

**TABLE 3:** Univariate ordinal polytomous logistic regression analysis.

	Hepatic inflammation		Hepatic fibrosis	
	OR (95% CI)	*p*-value	OR (95% CI)	*p*-value
Age (years)	1.017 (0.970–1.067)	0.489	1.111 (1.057–1.168)	<0.001
Female sex	1.432 (0.969–2.122)	0.072	1.086 (0.722–1.637)	0.691
Hypertension	0.931 (0.634–1.367)	0.717	1.300 (0.878–1.928)	0.190
Pattern of liver damage				
Hepatocellular	3.110 (2.050–4.749)	<0.001	Reference	
Cholestatic	Reference		1.932 (1.268–2.959)	0.002
Mixed	2.066 (1.320–3.247)	0.002	1.939 (1.175–3.215)	0.01
PLT (×10^9^/L)	0.996 (0.994–0.999)	0.009	0.990 (0.987–0.993)	<0.00
AST (U/L)	1.003 (1.002–1.004)	<0.001	1.000 (0.999–1.001)	0.82
TBIL (μmol/L)	1.009 (1.006–1.012)	<0.001	1.001 (0.998–1.003)	0.62
Autoimmunity	2.058 (1.444–2.942)	<0.001	1.856 (1.291–2.678)	0.001

PLT, platelet; AST, aspartate aminotransferase; TBIL, total bilirubin.

**FIGURE 4:**
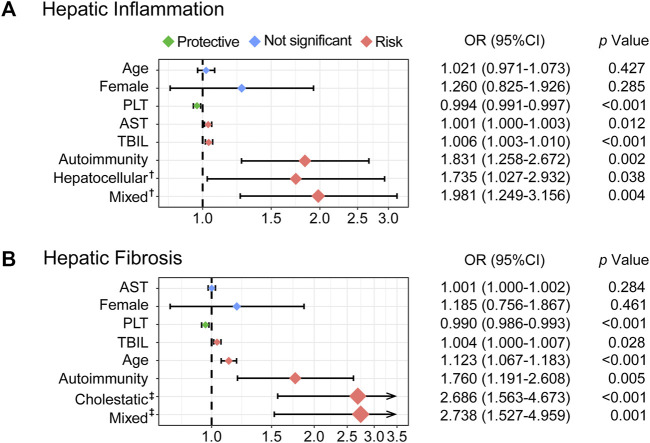
Forest plots based on multivariable ordinal polytomous logistic regression analysis. The *X*-axis was transformed as a log scale **(A)**. The risk factors associated with hepatic inflammation **(B)**. The risk factors associated with hepatic fibrosis. The colored solid diamonds represent the OR and the black lines show 95% CI.OR, odds ratio; CI, confidence interval; AST, aspartate transaminase; TBIL, total bilirubin; PLT, platelet.

Taking the cholestatic damage pattern as a reference, hepatocellular (OR: 1.735, 95% CI: 1.027–2.932, *p* = 0.038) and mixed (OR: 1.981, 95% CI: 1.249–2.932, *p* = 0.004) damage were independent risk factors for hepatic inflammation. However, cholestasis (OR: 2.686, 95% CI: 1.563–4.673, *p* < 0.001) and mixed (OR: 2.738, 95% CI: 1.527–4.959, *p* = 0.001) were independent risk factors for hepatic fibrosis when referring to the hepatocellular damage pattern. In addition, sex was not associated with either aspect (Figure 4).

## Discussion

With the aging of the population, the incidence of elderly DILI is expected to increase (Andrade et al., 2019a). Meanwhile, DILI causes the progression of underlying diseases due to drug withdrawal, a worse quality of life, and an immense economic burden (Stevens and Baker, 2009; Kullak-Ublick et al., 2017). We revealed that the presence of autoimmunity was associated with a higher level of illness severity concerning hepatic inflammation and fibrosis in elderly patients with DILI, providing a reference for the strategy of intensive monitoring and aggressive treatment.

DILI is an invisible killer, and although most cases are improved after drug withdrawal (Aithal et al., 2011; Chen et al., 2011), serious clinical outcomes such as chronic hepatitis, liver failure, cirrhosis, liver transplantation, or even death may occur if long-term drug use is combined with irregular liver function reexamination (Raschi and De Ponti, 2017; Weaver et al., 2020; Wang et al., 2021). In the USA and Europe, DILI accounts for the most cases of acute liver failure (Ostapowicz, 2002; Kullak-Ublick et al., 2017). A prospective study showed that patients aged over 60 years with comorbidities had a higher mortality rate (Chalasani et al., 2015). A retrospective analysis including 595 patients demonstrated that the incidence of chronic hepatitis, liver failure, cirrhosis, and death was 13.4%, 7.9%, 7.6%, and 4.5% in China, respectively (Zhu et al., 2016). The present study showed that 9.8% of patients presented cirrhosis, implying that the adverse outcome of DILI, especially in elderly individuals, might be underestimated and need more attention.

Our study found that the most commonly-used implicated drug in the elderly was cardiovascular drugs, which was different from the ordinary patient population. Previous studies demonstrated antibiotics to be the most commonly implicated agents (Andrade et al., 2019b). This might lie in the dominance of cardiovascular disease as an underlying disease in elderly individuals. Additionally, the elderly population was also found to be exposed to greater usage of over-the-counter drugs and herbal supplements due to the increased consciousness about health in the present study. Moreover, some features in the medication of the elderly should be noted: 1) higher level of polypharmacy usage, 2) more cases of unclear medication duration, 3) longer duration of drug intake until the onset of DILI, and 4) more cases of the cholestatic pattern of liver damage, which should not be neglected before the prescription.

Regarding clinical characteristics, 73.5% of participants were female, and the presence of autoimmunity was found to be more common in specimens from female patients, which is consistent with previous studies (Amacher, 2014; Chalasani et al., 2015) suggesting that females are more susceptible to DILI and autoimmunity. Levels of immunoglobulins and autoantibodies are higher in female, especially, postmenopausal women with DILI and abnormal IgG levels are more likely to progress autoimmune hepatitis (Sakiani et al., 2013; He et al., 2022). This may be attributed to immune function impacted by periodicity of estrogen and progesterone levels (Moulton, 2018). In our study, the elderly female patients were all postmenopausal women with a great potential for autoimmunity. Moreover, sex differences in percentage of body fat, cytochrome P450 isozymes and drug-binding proteins may also explain this phenomenon (Amacher, 2014). The cholestatic (47.6%) pattern was the main pattern of liver damage. Likewise, Lucena et al. (2009) proposed that agedness is positively associated with cholestatic liver injury. This may be explained by the high utilization rate of hyperlipophilic drugs and decreased biliary function (Hunt et al., 2014). Additionally, prolonged tubular excretion and cholangiocyte exposure may be the reason for activating immune responses (Weersink et al., 2021).

There was no statistically significant difference in hepatic inflammation or fibrosis for comorbidities, such as hypertension, hyperlipidemia, diabetes mellitus, or coronary heart disease. A Spanish DILI registry study suggested that hypertension and diabetes may be detrimental to the repair of liver injury, contributing to chronic outcomes (Medina-Caliz et al., 2016). Weersink et al. (2021) showed increasing comorbidity burden (*p* < 0.001) and polypharmacy (*p* < 0.001) in elderly patients with DILI, which may explain the increased non-liver-related mortality (*p* = 0.030). It may be that comorbidities are not direct factors contributing to the illness progression of DILI but make patients forced to take multiple drugs or actively seek informal medical methods such as folk remedies or dietary supplements.

Furthermore, according to the multivariate ordinal polytomous logistic regression analysis, autoimmunity was an independent risk factor for hepatic inflammation (OR: 1.831; 95% CI: 1.258–2.672) or fibrosis (OR: 1.760; 95% CI: 1.191–2.608), promoting illness progression in elderly DILI patients. Moreover, DILI with autoimmunity was not rare, accounting for 45.6% of cases, in the present study. An analysis of the autoimmune features of DILI from the DILI Network prospective study found that the majority of patients (60%–70%) were positive for ANA and SMA, and approximately 40% had elevated IgG serum levels (de Boer et al., 2017). Multidrug compatibility in the elderly can alter the immune and inflammatory response environment (Chen et al., 2015). Damage-associated molecular patterns (DAMPs) released by drug-injured hepatocytes activate innate immunity and lead to sterile inflammation, which further amplifies tissue damage (Mosedale and Watkins, 2017; Gerussi et al., 2021). Once more, inappropriate maturation of dendritic cells due to tissue injury in the elderly may alter the balance between immune function and tolerance, triggering the propensity of autoimmunity (Agrawal et al., 2012; Tajiri and Shimizu, 2013). Despite the application of high-dose glucocorticoids in the follow-up, drug-mediated autoimmune hepatitis mostly occurrs in elderly patients (75% aged >60 years) predisposed to late relapse (Yeong et al., 2016). Thus, our conclusion that autoimmunity promotes the illness progression of elderly DILI is of significant clinical manfulness.

Therefore, elderly DILI patients with autoimmunity need more frequent follow-up and aggressive treatment. Over the years, several models (Ashby et al., 2021; Li et al., 2021) have been proposed to predict the outcome of DILI. Our previous study found that significant hepatic inflammation (HAI ≥10) was an independent risk factor for biochemical resolution (OR: 21.278, 95% CI: 14.780, 30.632) (Wang et al., 2022), which was consistent with the present result.

The primary treatment for DILI is the discontinuation of suspected drugs, and corticosteroids can improve the condition of DILI with autoimmune features (Chalasani et al., 2021; Björnsson et al., 2022). Nevertheless, clinicians must weigh the risks of infection, osteoporosis, cognitive decline, etc., against the progression of DILI when treating older adults with corticosteroids (Lee et al., 2021).

The strengths of the present study included that: 1) a multilevel ordinal logistic regression analysis was performed to obtain a reliable estimate and standard error, 2) a large sample size of elderly patients was enrolled, which had adequate power to detect the true effect of the independent variables, and 3) a liver biopsy was required to ensure the accurate assessment for hepatic inflammation and fibrosis and exclusion of patients with other etiologies, which is scarce in the current literature.

Although the findings have important clinical implications, several limitations should be noted. First, the cross-sectional study design cannot establish the causal relationship between the severity of DILI (hepatic inflammation or fibrosis) and identified independent variables (e.g., autoimmunity, patterns of injuries, etc.). Second, implicated agents might be misclassified due to the multiple underlying diseases and complexity of drug use. Finally, as this study is a retrospective single-center study, there might be some biases, such as admission rate bias. There were no patients of other races, which might limit the generalizability of this conclusion to broader populations. Further multicenter prospective studies with a larger sample size are warranted to verify the results.

## Conclusion

Female sex and the cholestatic liver damage pattern are dominant in the elderly patients with DILI, and comorbidities are not directly associated with the severity of the illness. The presence of autoimmunity represents a more serious illness state of DILI, deserving more intensive monitoring and progressive treatment.

## Data Availability

The raw data supporting the conclusion of this article will be made available by the authors, without undue reservation.

## Abbreviations

ANA, anti-nuclear antibody; AMA, anti-mitochondrial antibody; ALT, Alanine aminotransferase; AST, Aspartate aminotransferase; ALP, Alkaline phosphatase; BMI, body mass index; CI, confidence interval; DILI, drug-induced liver injury; EASL, uropean association for the study of the liver; HDS, herbal and dietary supplements; HIV, human immunodeficiency virus; HAI, histological activity index; IgG, immunoglobulin G; IQR, interquartile range; NSAID, non-steroidal anti-inflammatory drug; OR, odds ratio; PLT, platelet; PIM, potentially inappropriate medications; RUCAM, roussel uclaf causality assessment method; SMA, smooth muscle antibody; TBIL, total bilirubin; TCM, traditional Chinese medicine; ULN, upper limit of normal.
