# Supplementary material for: Autoimmunity associates with severity of illness in elderly patients with drug-induced liver injury
Source: Front Pharmacol. 2023 Feb 16;14:1071709. doi: 10.3389/fphar.2023.1071709 (PMC9978525; doi:10.3389/fphar.2023.1071709)
Supplement: Supplementary file 1 [file DataSheet1.docx]

Autoimmunity Associates with Severity of Illness in Elderly Patients with Drug-Induced Liver Injury

**SUPPLEMENTARY FIGURE |** The percentage of implicated drugs.


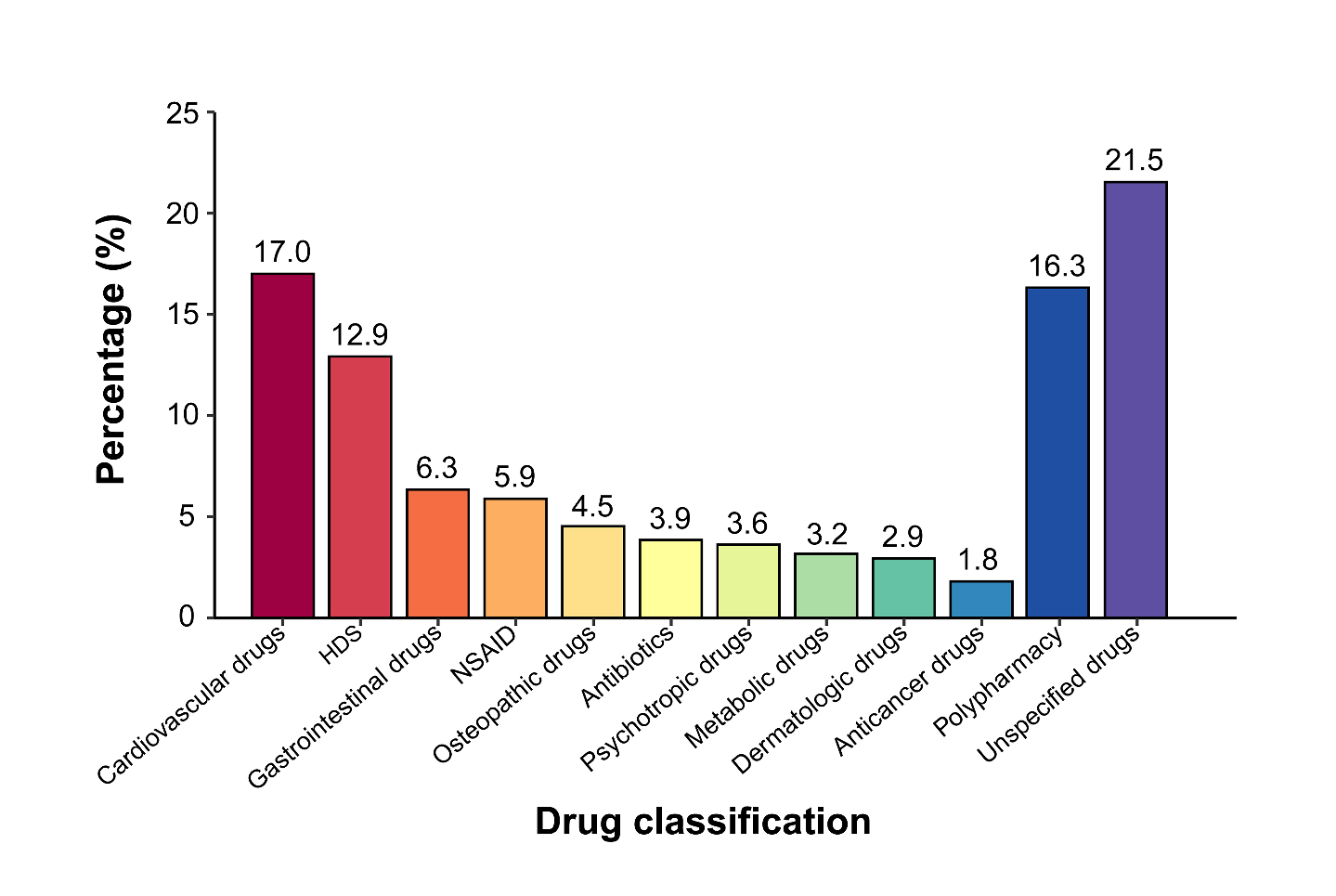


*HDS, herbal and dietary supplements; NSAID, non-steroidal anti-inflammatory drug.*
